# MV–MR: Multi-Views and Multi-Representations for Self-Supervised Learning and Knowledge Distillation

**DOI:** 10.3390/e26060466

**Published:** 2024-05-29

**Authors:** Vitaliy Kinakh, Mariia Drozdova, Slava Voloshynovskiy

**Affiliations:** Department of Computer Science, University of Geneva, 1227 Carouge, Switzerland; vitaliy.kinakh@unige.ch (V.K.); mariia.drozdova@unige.ch (M.D.)

**Keywords:** image representation learning, self-supervised learning, knowledge distillation, semi-supervised learning

## Abstract

We present a new method of self-supervised learning and knowledge distillation based on multi-views and multi-representations (MV–MR). MV–MR is based on the maximization of dependence between learnable embeddings from augmented and non-augmented views, jointly with the maximization of dependence between learnable embeddings from the augmented view and multiple non-learnable representations from the non-augmented view. We show that the proposed method can be used for efficient self-supervised classification and model-agnostic knowledge distillation. Unlike other self-supervised techniques, our approach does not use any contrastive learning, clustering, or stop gradients. MV–MR is a generic framework allowing the incorporation of constraints on the learnable embeddings via the usage of image multi-representations as regularizers. The proposed method is used for knowledge distillation. MV–MR provides state-of-the-art self-supervised performance on the STL10 and CIFAR20 datasets in a linear evaluation setup. We show that a low-complexity ResNet50 model pretrained using proposed knowledge distillation based on the CLIP ViT model achieves state-of-the-art performance on STL10 and CIFAR100 datasets.

## 1. Introduction

Self-supervised learning (SSL) methods are alternatives to supervised ones. In recent years, the gap between SSL and supervised methods has decreased in performing downstream tasks, including image classification [1], object detection [2], and semantic image segmentation [3,4]. A general idea behind the SSL models for image classification is to train an embedding network, often called an *encoder*, on an unlabeled dataset and then to use this pretrained encoder for the downstream tasks.

The general goal is to ensure the invariance of embeddings to different inputs known as *augmentations* or *views.* However, this approach might lead to trivial solutions when two branches of encoders produce the same output. As a result, one observes an effect known as a *collapse* in training when no meaningful representation can be learned for different inputs. Therefore, there have been a lot of recent works tackling this issue by regularizing the networks to avoid such a collapse. Several key approaches have been developed to mitigate these negative impacts, using different tactics. The first group of methods aims to directly maximize the mutual information between the input image x and its positive pairs created on the basis of augmentations. In this view, an exponential prior on the conditional distribution in the representation space and an associated contrastive loss with positive–negative pairs as in InfoNCE [5] is assumed. Unfortunately, such an approach is quite computationally expensive in practice, due to the need for a large batch size to incorporate the large number of negative pairs. The second group of methods aims to avoid collapse by introducing different asymmetries in two branches at the training stage. Examples of this approach are training one network with gradient descent and updating the other with an exponential moving average of the weights of the first network [6] or introducing regularizers on the learned representations such as regularization by decorrelation on the dimensions of the embeddings [7], etc. The third group of methods is Masked Image Modeling (MIM). These methods primarily focus on avoiding collapse and learning rich image representations by predicting the missing parts in masked inputs. This methodology relies on masking a portion of the input image and training the model to predict these masked parts, thereby learning contextual and semantic information. A notable method in this domain is the BEiT [8], which introduces a transformer-based model that learns to predict the masked visual tokens, analogous to the masked language modeling in NLP. Another significant approach is the MAE (Masked Autoencoder) [9], which uses an asymmetric encoder–decoder structure, where the encoder processes only visible patches and the decoder reconstructs the masked patches. While MIM effectively learns representations, it is a transformer-specific approach and not transferable to other architectures.

The proposed approach avoids the embeddings’ collapse by introducing the dependence maximization between trainable embeddings and hand-crafted features using *distance correlation* [10]. Distance correlation, unlike other losses in latent space, allows computing dependencies between feature vectors of different shapes. We maximize the dependence between different embeddings while preserving the variance in them. We show that variance preservation maximizes the entropy of embeddings, which makes them unique and distinguishable. Our approach is different from InfoNCE [5], which advocates a contrastive loss that maximizes the mutual information (MI) between input image x and its positive pairs. In contrast to the InfoNCE, our approach is not contrastive, does not require large batch sizes, and allows computing the distance between embeddings and features of any shape. It is also different from methods such as Barlow Twins [7] and VICReg [11] since we do not explicitly minimize the dependencies between the components within the embedding.

We also show that the proposed approach can be used for efficient representation learning and latent space-agnostic knowledge distillation. The approach is based on the dependence maximization between the embeddings of the target trainable encoder, represented by the ResNet50 [12], and the embeddings of the pretrained encoder, represented by the CLIP [13] (based on ViT-B-16 [14]). Since the distance correlation is agnostic to the latent space shape, any pretrained encoder with any latent space can be used for knowledge distillation. To our best knowledge, we are the first to propose a model-distillation method that is agnostic to the latent space shape.

The main goal behind MV–MR is twofold: (i) maximizing the invariance of embeddings, i.e., maximizing the proximity of embeddings for the same image observed under different views, and (ii) maximizing the amount of information in each embedding, i.e., maximizing the variability of the embedding. Furthermore, to avoid the collapse during training, we regularize the branch with the augmentations by imposing the dependence constraints on a set of representations extracted from various encodings.

The proposed approach introduces several unique features: (i) we introduce a novel SSL approach that avoids collapse thanks to an additional regularization term that maximizes the dependence between trainable embeddings and various feature vectors using distance correlation; (ii) up to our best knowledge, the proposed method is among the first that uses the dependence maximization of the latent space based on distance correlation for SSL; (iii) the proposed method is agnostic to the latent space shape and, thus, can be used with any types of features; (iv) we introduce a novel knowledge distillation technique that is agnostic to model and shape of latent space; (v) we demonstrate the state-of-the-art classification results on the STL10 [15] (89.71%) and CIFAR20 [16] (73.2%) datasets using a linear evaluation protocol for non-contrastive SSL methods; (vi) we provide the information-theoretic explanation of the proposed method that contributes to the explainable ML; (vii) we demonstrate how the complex CLIP model with 86.2 M parameters trained on 400 M text–image pairs can be distilled into a ResNet50 model with just 23.5 M parameters trained on the STL10, CIFAR100 [17], and ImageNet-1k [18] datasets; (viii) we achieve state-of-the-art performance in knowledge distillation in the image-classification task using the ResNet50 model as student and CLIP ViT-B-16 as teacher on CIFAR100, with **78.6%** accuracy.

We have three loss terms in our objective function: (a) the first term $L_{1}$ consists of the mean square error (MSE) loss between the embeddings from the non-augmented view and augmented views of the same image; it is used for the invariance of embeddings, and we introduce additional variation terms that are used for maximization of the variability of the embeddings (we demonstrate that this term originates from an upper bound on mutual information between these embeddings under corresponding assumptions); (b) the second term $L_{2}$ stands for the distance correlation between the embeddings from the augmented and non-augmented views that complements the first term to capture non-linear relations between the embeddings; and (c) the third term $L_{3}$ corresponds to the distance correlation between the embeddings from the augmented view and multiple image representations. For the non-learnable or hand-crafted representations, we have studied various techniques of invariant data representation that are well-known in computer vision and image-processing applications. The studied hand-crafted features include, but are not limited tp, ScatNet [19] features, local standard deviation (LSD)-based [20] filters, and histograms of oriented gradients (HOG) [21]. Additionally, to demonstrate the flexibility of the proposed method, we have also considered random augmentations of the original images flattened into feature vectors as instances of hand-crafted features. Since distance correlation is shape-agnostic for the features, we are able to combine features of different shapes in the loss functions. Also, replacing hand-crafted features with embeddings from pretrained networks is used for model distillation, without the need to change losses, architecture, or feature dimensionality.

## 2. MV–MR: Motivation and Intuition

**Figure 1:**
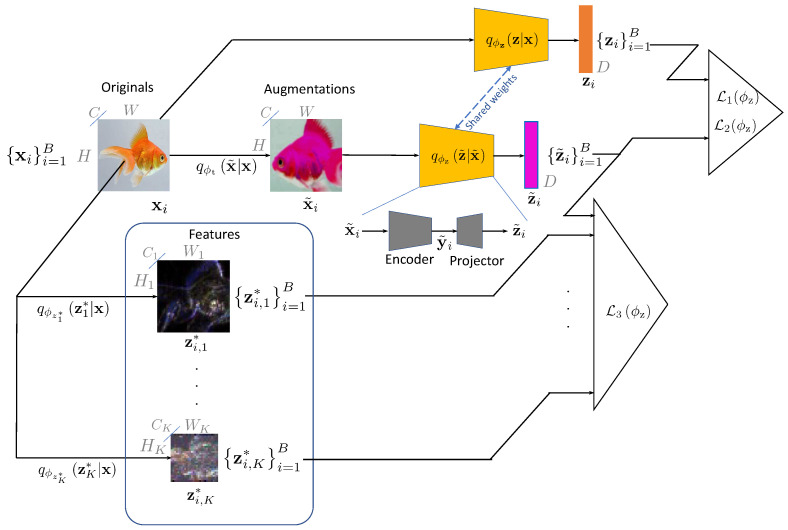
MV–MR: proposed SSL approach. Two *views* of the image are produced: one original and the other augmented by $q_{\phi_{t}}\left(\tilde{x}|x\right)$. Then, this first view is encoded via encoder $q_{\phi_{z}}\left(z|x\right)$, producing $z_{i}$, which denotes an original embedding, and via $q_{\phi_{z}}\left(\tilde{z}|\tilde{x}\right)$, producing ${\tilde{z}}_{i}$, denoting an augmented one. The representations $z_{i,k}^{*}$ are obtained via *K* hand-crafted feature extraction mappers $q_{\phi_{z_{k}^{*}}}\left(z_{k}^{*}|x\right),1\leq k\leq K$. The same process is applied to each image $x_{i}$ in the batch 1≤i≤B. The embedding is regularized by a loss $L_{1}\left(\phi_{z}\right)$, minimizing the Euclidean distances between the embeddings $z_{i}$ and ${\tilde{z}}_{i}$ while ensuring that their variance is above a threshold. The loss $L_{2}\left(\phi_{z}\right)$ ensures the dependence between the pair of augmented and non-augmented embeddings using the distance correlation. The regularization loss $L_{3}\left(\phi_{z}\right)$ is imposed by maximizing the distance correlation between the augmented embedding ${\tilde{z}}_{i}$ and a set of hand-crafted features $z_{i,k}^{*},1\leq k\leq K$ computed for the given batch *B*.

**Figure 2:**
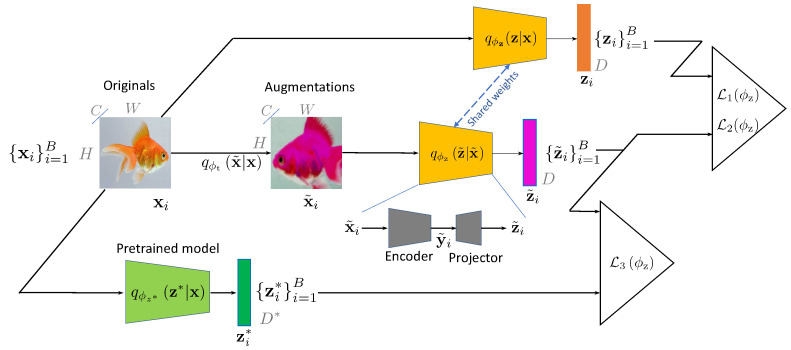
MV–MR: distillation approach. $q_{\phi_{z^{*}}}\left(z^{*}|x\right)$ is the high-complexity (in term of parameters) teacher model used as a feature extractor in order to train a low-complexity student model $q_{\phi_{z}}\left(z|x\right)$. The teacher model corresponds to a set of hand-crafted feature extractors in Figure 1. The representations $z_{i}^{*}$ are obtained from the pretrained teacher model $q_{\phi_{z^{*}}}\left(z^{*}|x\right)$. The same losses as in self-supervised pretraining are used: $L_{1}\left(\phi_{z}\right)$ minimizes the Euclidean distances between the embeddings $z_{i}$ and ${\tilde{z}}_{i}$ while ensuring that their variance is above a threshold, $L_{2}\left(\phi_{z}\right)$ ensures the dependence between the pair of augmented and non-augmented embeddings using the distance correlation, and $L_{3}\left(\phi_{z}\right)$ maximizes the distance correlation between the augmented embedding ${\tilde{z}}_{i}$ and the teacher’s embeddings $z_{i}^{*}$.

MV–MR pretraining and distillation schemes are schematically shown in Figure 1 and Figure 2, respectively. The dimensions of embeddings with and without augmentations are the same, i.e., $\tilde{z}\in R^{D}$ and $z\in R^{D}$, respectively. These embeddings are extracted from the augmented $\tilde{x}$ and non-augmented x via a generalized parametrized embedder $q_{\phi_{z}}\left(\cdot |\cdot \right)$ that can be deterministic or stochastic with parameters $\phi_{z}$. The encoder can be a parametrized neural network of any architecture. A $k^{th}$ hand-crafted descriptor $z_{k}^{*}$, where k∈{1,2,⋯,K} and *K* stands for the total number of hand-crafted descriptors, is generally a tensor of dimensions $H_{k}\times W_{k}\times C_{k}$ and is flattened to $D_{k}=H_{k}W_{k}C_{k}$. This descriptor is generally obtained via deterministic assignment $z_{k}^{*}=f_{\phi_{z_{k}^{*}}}\left(x\right)$ or sometimes via stochastic mapping $Z_{k}^{*}∼q_{\phi_{z_{k}^{*}}}\left(z_{k}^{*}|x\right)$, where $\phi_{{z^{*}}_{k}}$ denotes the parameters of the kth feature extractor.

### 2.1. Motivation: Regularization in Self-Supervised Representation Learning

The learned representation should contain the informative representation of data with lower dimensionality and should be invariant under some transformations, i.e., to ensure the same latent representation for the data from the same sample passed through certain transformations. The satisfaction of these conflicting requirements in practice is not a trivial task. Many state-of-the-art SSL techniques try to find a reasonable compromise between these requirements and practical feasibility solely in the scope of machine learning formulation by imposing certain constraints on the properties of the learned representation via the optimization of encoder parameters under augmentations.

At the same time, there exists a rich body of achievements in the computer vision community in the domain of the hand-crafted design of robust, invariant, yet discriminating data representations [21,22,23,24]. Generally, the computer vision descriptors are very rich in terms of targeted invariant features and quite efficient in terms of computation. However, to our best knowledge, such descriptors are not yet fully integrated into the development of SSL techniques. Therefore, one of the objectives of this paper is to propose a framework where the SSL representation learning might be coupled with the constraints on the embedding space offered by the invariant computer vision representations. Our objective is not to consider a case-by-case approach on how to couple SSL with a particular computer vision representation but instead to propose a *generic approach* where any form of desirable computer vision representation can be integrated into the SSL optimization problem in an easy and tractable way. This ensures that the learned representation possesses the targeted properties inherited from the used computer vision descriptors. Furthermore, features extracted by such descriptors might be considered as a form of invariant data representation, which is one of the desired properties of trained encoders. Thus, maximizing the dependence between the trainable embedding and such representation might be a novel form of regularization, leading to an increased-invariance yet collapse-avoiding technique. Since a single computer vision descriptor might not capture all desirable properties and have different representation formats, the targeted framework should be flexible enough to deal uniformly with all these descriptors within a simple optimization problem. Distance correlation is very useful for this kind of representation learning, since it allows one to incorporate features of any shapes, without the need to match the shape of learnable embeddings and hand-crafted target embeddings.

In summary, our motivation is to include regularization constraints on the solution by borrowing some inherent computer vision feature invariance to certain transformations. In this way, we target learning the low-dimensional embedding, which contains only essential information about the data that might be of interest for the targeted downstream task and where all information about the augmentations is excluded.

### 2.2. Intuition

The basic idea behind MV–MR is to introduce constraints on the invariance of embedding via a new loss function. Our overall objective is to maximize the mutual information $I(\tilde{Z};Z)$ between the augmented embedding $\tilde{Z}$ and the embedding without the augmentation Z and to maximize the mutual information $I(\tilde{Z};Z_{k}^{*})$ between $\tilde{Z}$ and some invariant feature $Z_{k}^{*}$ extracted from X using a mapper that ensures a known invariance to the desired transformation.

#### 2.2.1. Measuring Dependencies between Embeddings of Non-Augmented and Augmented Data

**Upper bound on mutual information**: In the first case, one can decompose the mutual information as

(1) $$\begin{array}{ccc} I(\tilde{Z};Z) & =E_{p(\tilde{z},z)}\left[\log\frac{p(\tilde{z},z)}{p\left(\tilde{z}\right)p\left(z\right)}\right] & =E_{p(\tilde{z},z)}\left[\log\frac{p(\tilde{z}|z)}{p(\tilde{z})}\right]\\ \; & =h\left(\tilde{Z}\right)-h\left(\tilde{Z}|Z\right), \end{array}$$

where $h\left(\tilde{Z}\right)=-E_{p(\tilde{z})}\left[\log p(\tilde{z})\right]$ denotes the differential entropy and $h\left(\tilde{Z}|Z\right)=-E_{p(\tilde{z},z)}$$\left[\log p(\tilde{z}|z)\right]$ denotes conditional differential entropy (we assume that the differential entropy is non-negative under the considered settings). Since the computation of the marginal distribution $p(\tilde{z})$ and conditional distribution $p(\tilde{z}|z)$ is difficult in practice, we proceed by bounding these terms. We assume that the desired embeddings need to be bounded by some variance $\sigma_{z}^{2}$ to avoid a training collapse when the encoders produce constant and non-informative vectors so that the entropy-maximizing distribution for the first entropy term is the Gaussian one, i.e., $p\left(\tilde{z}\right)\propto \frac{\exp\left(-\frac{1}{2}{\tilde{z}}^{T}\Sigma_{z}^{-1}\tilde{z}\right)}{\sqrt{{\left(2\pi \right)}^{D}\left|\Sigma_{z}\right|}}$, where $\Sigma_{z}$ represents the covariance matrix.

The conditional entropy $h(\tilde{Z}|Z)$ is minimized when the embedding $\tilde{Z}$ contains as much information as possible about Z, i.e., when two vectors are dependent. Assuming that $p\left(\tilde{z}|z\right)\propto \frac{1}{C_{z}}\exp\left(-\beta_{z}d\left(\tilde{z},z\right)\right)$, where $d(\tilde{z},z)$ denotes some distance between two vectors such as the $\ell_{2}$-norm for the Gaussian distribution or $\ell_{1}$-norm for the Laplacian one, where $C_{z}$ stands for the normalization constant and $\beta_{z}$ denotes a scaling parameter. Thus, the minimization of the conditional entropy $h(\tilde{Z}|Z)$ reduces to the minimization of the distance $d(\tilde{z},z)$.

**Distance covariance**: Another way to measure the dependency between the data is based on distance covariance, as proposed by [10]. In the general case of dependence between the data, the distance covariance is non-invariant to strictly monotonic transformations, unlike mutual information. Nevertheless, the distance covariance has several attractive properties: (i) it can be efficiently computed for two vectors that have generally different dimensions $\tilde{z}\in R^{D}$ and $z\in R^{D^{\prime }}$, such that $D\neq D^{\prime }$, and (ii) it is easier to compute in practice in contrast to the mutual information. Additionally, the distance covariance captures higher-order dependencies between the data, in contrast to the Pearson correlation. The *distance covariance* ${\mathrm{dCov}}^{2}\left(\tilde{Z},Z\right)$, proposed by [10], is defined as

(2) $${\mathrm{dCov}}^{2}\left(Z,\tilde{Z}\right)=\frac{1}{c_{D}c_{D^{\prime }}}\int_{R^{D+D^{\prime }}}\frac{{\left|\varphi_{Z,\tilde{Z}}\left(t,u\right)-\varphi_{Z}\left(t\right)\varphi_{\tilde{Z}}\left(u\right)\right|}^{2}}{{\left|t\right|}_{D}^{1+D}{\left|u\right|}_{D^{\prime }}^{1+D^{\prime }}}dtdu,$$

which measures the distance between the joint characteristic function $\varphi_{Z,\tilde{Z}}\left(t,u\right)$ and the product of the marginal characteristic functions $\varphi_{Z}\left(t\right)\varphi_{\tilde{Z}}\left(u\right)$ [10]. This definition has a lot of similarities to the mutual information in (1), which measures the ratio between the joint distribution $p(\tilde{z},z)$ and the product of marginals $p\left(\tilde{z}\right)p\left(z\right)$. Since $\varphi_{Z,\tilde{Z}}\left(t,u\right)=\varphi_{Z}\left(t\right)\varphi_{\tilde{Z}}\left(u\right)$ when $\tilde{Z}$ and Z are independent random vectors, the distance covariance is equal to zero.

In the following, we proceed with the normalized version of distance covariance, known as *distance correlation*, defined as

(3) $$\mathrm{dCor}\left(\tilde{Z},Z\right)=\frac{{\mathrm{dCov}}^{2}\left(\tilde{Z},Z\right)}{\sqrt{\mathrm{dVar}\left(\tilde{Z}\right)\mathrm{dVar}\left(Z\right)}},$$

where $0\leq \mathrm{dCor}(\tilde{Z},Z)\leq 1$ and $\mathrm{dVar}\left(Z\right)={\mathrm{dCov}}^{2}\left(Z,Z\right)$.

*Sample distance covariance*, for a given $Z_{B}=\left[z_{1},\ldots ,z_{B}\right]$, denoting a batch of size *B* of embeddings from original views, and ${\tilde{Z}}_{B}=\left[{\tilde{z}}_{1},\ldots ,{\tilde{z}}_{B}\right]$, referring to a batch of embeddings from augmented views, is defined as

(4) $${\mathrm{dCov}}_{B}^{2}\left({\tilde{Z}}_{B},Z_{B}\right):=\frac{1}{B^{2}}\sum_{j=1}^{B}\sum_{i=1}^{B}A_{j,i}C_{j,i}.$$

In Equation (4), we use the notations $A_{j,i}:=a_{j,i}-{\bar{a}}_{j\cdot }-{\bar{a}}_{\cdot i}+{\bar{a}}_{\cdot \cdot },\;$$C_{j,i}:=c_{j,i}-{\bar{c}}_{j\cdot }-{\bar{c}}_{\cdot i}+{\bar{c}}_{\cdot \cdot }$, where $a_{j,i}=∥{\tilde{Z}}_{B_{j}}-{\tilde{Z}}_{B_{i}}∥,\;$$c_{j,i}=∥Z_{B_{j}}-Z_{B_{i}}∥$, where j,i=1,2,…,B. Finally, *sample distance correlation* is defined as:

(5) $${\mathrm{dCov}}_{B}\left({\tilde{Z}}_{B},Z_{B}\right)=\frac{{\mathrm{dCov}}_{B}^{2}\left({\tilde{Z}}_{B},Z_{B}\right)}{\sqrt{{\mathrm{dVar}}_{B}\left({\tilde{Z}}_{B}\right){\mathrm{dVar}}_{B}\left(Z_{B}\right)}},$$

with ${\mathrm{dVar}}_{B}\left(Z_{B}\right)={\mathrm{dCov}}_{B}^{2}\left(Z_{B},Z_{B}\right)$.

#### 2.2.2. Dependence between Embeddings of Augmented Data and Multiple Hand-Crafted Representations

The second mutual information $I(\tilde{Z};Z_{k}^{*})$ between $\tilde{Z}$ and some invariant feature $Z_{k}^{*}$ deals with vectors of different dimensions. Thus, one can either map these vectors to the same dimension and apply the above arguments, use the Hilbert–Schmidt proxy [25], or proceed with the distance correlation dependence measure for the uniformity of consideration. We focus on the distance correlation case due to its property of handling vectors of different dimensions and its ability to capture higher-order data statistics.

## 3. Related Work

**Pretext task methods.** The main idea behind these methods is to design a specific task, *a.k.a. pretext task*, for the dataset that contains some “labels” of the pretext task without having any access to the labels of the target task. Such pretext tasks include, but are not limited to, applying and predicting parameters of the geometric transformations [26], jigsaw puzzle solving [27], inpainting [28] and colorization [29] of the images, and reversing augmentations. Typically, the pretext task methods have been coupled with other SSL techniques in recent years [30,31,32].

**Contrastive methods.** Most of the contrastive SSL methods are based on different extensions of the InfoNCE [5] formulation. The InfoNCE method is based on the direct maximization of the mutual information between the input image and its positive pairs via minimization of the contrastive loss. Examples of contrastive methods are SimCLR [33], SwAV [34], and DINO [35].

**Clustering methods.** Clustering-based SSL methods are based on the idea of assigning cluster labels to the learned representations in an unsupervised manner with some regularization, such as maintaining uniformity of these cluster labels. The DeepCluster [36] method iteratively groups the features from the encoder using the standard *k*-means clustering and then uses them as an assignment for the supervision to update the weights of the encoder at the next iterations. SwAV [34] and DINO [35] are other notable clustering-based SSL methods that combine contrastive learning and clustering by clustering the data while requiring the same cluster assignment for different views of the same image.

**Distillation methods.** Distillation-based SSL methods like BYOL [37], SimSiam [6], and others use the teacher–student type of training, where the student network is trained with the gradient descent, while the teacher network is not updated with gradient descent, but rather with an exponential moving-average update or other method. Such a design is used to avoid collapse.

**Collapse- preventing methods.** Similar to distillation, collapse-preventing methods try to prevent the collapse by the usage special regularization of embeddings. The Barlow Twins [7] method aims to make the covariance matrix of the embeddings to be an identity matrix. This means that each dimension of the embeddings should be decorrelated with all other dimensions. Additionally, the minimum variance of embedding per each dimension in the batch is constrained. The VICReg [11] method extends the Barlow Twins [7] approach by imposing an additional constraint on the distance between the embeddings with and without augmentations.

**Masked Image Modeling.** Masked Image Modeling (MIM) for self-supervised learning has emerged as a compelling approach, diverging from traditional methods like pretext task, contrastive, or clustering methods. Central to MIM is the principle of intentionally masking portions of an input image and training a model to predict these occluded parts. This process enables the model to learn valuable representations of the data without relying on explicit labels. Unlike contrastive learning methods like SimCLR or SwAV that require negative samples, MIM directly utilizes the spatial coherence of images to enhance the model’s ability to recognize and predict the structure within masked areas. Pioneering examples include the BEiT [8] algorithm, which employs a transformer architecture to predict the masked visual tokens, drawing inspiration from masked language modeling. Another notable implementation is the MAE (Masked Autoencoder) [9], which uses an asymmetric encoder–decoder structure to efficiently reconstruct masked patches. These approaches contrast with distillation methods like BYOL, where a teacher–student model is used, and clustering methods like DeepCluster that focus on feature clustering. MIM’s uniqueness lies in its direct engagement with the raw image data, offering a pathway to learn intricate image features in a self-supervised manner without the need for complex negative sample handling or clustering mechanisms.

**Knowledge distillation.** Knowledge distillation [38] is a type of model optimization, where a simple small model (student model) is trained to match the bigger complex model (teacher model). There are multiple types of knowledge-distillation schemes: offline distillation [39], online distillation [40], and self-distillation [41]. There are multiple types of knowledge types that are used for distillation: response-based knowledge [39,42], feature-based knowledge [43], and others. We show how our method can be used for offline feature-based knowledge distillation.

## 4. MV–MR: Detailed Description

### 4.1. Method

The training objective consists of two parts: (a) ensuring the invariance of the learned representation under the applied augmentations and, simultaneously, (b) imposing constraints on the learned representation. The final loss integrates the loss based on the upper bound of the mutual information and distance correlation.

#### 4.1.1. Training Objectives for the Representation Learning Based on the Mutual Information

We follow the overall idea of ensuring the invariance of learned representation under the family of applied augmentations and we proceed along the line discussed in the previous section. Since both branches have the same dimension *D*, we proceed with the maximization of the upper bound on the mutual information between these dimensions, as considered in Section 2.2.1.

To train the deterministic encoder $q_{\phi_{z}}\left(z|x\right)=\delta \left(z-f_{\phi }\left(x\right)\right)$, we penalize the embeddings ${\tilde{z}}_{i}$ of augmented view ${\tilde{x}}_{i}$ to be as close as possible to the embeddings $z_{i}$ of non-augmented view $x_{i}$ using the MSE loss between them:

(6) $$d\left(Z_{B},{\tilde{Z}}_{B}\right)=\frac{1}{B}\sum_{i=1}^{B}{∥z_{i}-{\tilde{z}}_{i}∥}_{2}^{2}.$$

The MSE is frequently used to ensure the similarity between embeddings. It can be demonstrated that this term equates to the conditional entropy term in the mutual information, as specified in Equation (1), assuming Gaussian conditional distribution. At the same time, it can be proven that InfoNCE aims to minimize the negative cross-entropy $h(\tilde{Z}|Z)$ while maximizing the entropy h(Z) for the entropy-based model parametrization of $p(\tilde{z}|z)$, with $p\left(\tilde{z}\right)=E_{p(z)}\left[p(\tilde{z}|z)\right]$. Hence, the MSE loss is a non-contrastive loss based on $h(\tilde{Z}|Z)$, while InfoNCE operates as its contrastive counterpart.

The variance-regularization term corresponding to the entropy term in the mutual information in (1) is used to control the variance of the embeddings. We use a hinge function of the standard deviation of the embeddings along the batch dimension:

(7) $$v\left(Z_{B}\right)=\frac{1}{D}\sum_{d=1}^{D}\max\left(0,\gamma -S\left(z[d],\epsilon \right)\right),$$

where z[d] denotes the dth dimension of z, γ is the margin parameter for the standard deviation, ϵ is a small scalar for numerical stability, and *S* is the standard deviation, defined as

(8) $$S\left(a,\epsilon \right)=\sqrt{\mathrm{Var}(a)+\epsilon }.$$

We define the loss that ensures the correspondence between the embeddings from the augmented and non-augmented views, i.e., positive pairs. Simultaneously, we bound the variance of both embeddings as follows:

(9) $$L_{1}\left(\phi_{z}\right)=\lambda d\left(Z_{B},{\tilde{Z}}_{B}\right)+\mu \left[v\left(Z_{B}\right)+v\left({\tilde{Z}}_{B}\right)\right],$$

where λ and μ are hyper-parameters controlling the importance of each term in the loss, $z=f_{\phi_{z}}\left(x\right)$ and $\tilde{z}=f_{\phi_{z}}\left(\tilde{x}\right)$. We set λ and μ to 1 in our experiments. This loss is parametrized by the parameters $\phi_{z}$ of the encoder and projector. It should be pointed out that, for the symmetry, we impose the constraint on the variance for both augmented and non-augmented embeddings.

It is interesting to point out that the obtained result coincides with the loss used in VICReg [11], where its origin was not considered, from the information-theoretic point of view, as the maximization of mutual information between the embeddings. At the same time, it should be noted that there is no constraint on the covariance matrix of embeddings as in the VICReg [11] and Barlow Twins [7] methods.

#### 4.1.2. Training Objectives for Representation Learning Based on Distance Covariance

The distance correlation is used for the dependence maximization between the embedding from the augmented view with the non-augmented one and the set of representations from the hand-crafted functions.

Accordingly, the loss $L_{2}\left(\phi_{z}\right)$ denotes the minimization formulation of the distance correlation maximization problem between embeddings from augmented and non-augmented views:

(10) $$L_{2}\left(\phi_{z}\right)=\alpha \left[1-{\mathrm{dCor}}_{B}\left({\tilde{Z}}_{B},Z_{B}\right)\right],$$

and the loss $L_{3}\left(\phi_{z}\right)$ denotes the same for the embedding from the augmented view and kth hand-crafted representation (in the case of self-supervised pretraining) or embeddings from pretrained models (in the case of knowledge distillation):

(11) $$L_{3}\left(\phi_{z}\right)=\sum_{k=1}^{K}\beta_{k}\left[1-{\mathrm{dCor}}_{B}\left({\tilde{Z}}_{B},Z_{B_{k}^{*}}\right)\right],$$

where α and $\beta_{k}$ are hyper-parameters controlling the importance of each term in the loss. In our experiments, we set α=1 and $\beta_{k}=1$.

The final loss function is a sum of three losses:

(12) $$L\left(\phi_{z}\right)=L_{1}\left(\phi_{z}\right)+L_{2}\left(\phi_{z}\right)+L_{3}\left(\phi_{z}\right).$$

## 5. Applications of the Proposed Model

In this section, we demonstrate the application of the proposed model to (a) self-supervised pretraining of the model for classification and (b) self-supervised model distillation.

### 5.1. Self-Supervised Pretraining for Classification

The proposed method can be used for efficient self-supervised model pretraining for classification. Once pretrained, the model is finetuned for classification. We report our results on the STL10 [15] and ImageNet-1K [18] datasets on linear evaluation and semi-supervised finetuning, with 1% and 10% of label pipelines in Table 1 and Table 2. In a linear evaluation pipeline, the pretrained encoder is used as is, without further training, while only a one-layer linear classifier is trained with labeled data. In the semi-supervised finetuning pipeline, the classifier head is attached to the pretrained encoder and the full model is finetuned on the labeled data.

### 5.2. Knowledge Distillation

The proposed method can also be used for efficient knowledge distillation. This is performed by using the pretrained model (teacher) as the feature extractor and computing the distance correlation between the embeddings of the trainable encoder (student) and the pretrained teacher encoder. In practice, this can be used to match the performance of the big pretrained models with smaller models or match the performance of the models that have been trained on proprietary datasets. In contrast to the standard knowledge distillation approaches [39], our approach does not use any labels or require a latent space of the same shape. As a practical example, we demonstrate that, by using the proposed knowledge distillation approach, we are able to match the performance of the CLIP [13] based on ViT-B-16 [14] with 86.2 M parameters pretrained on 400 M images from the LAION-400M [44] dataset, using ResNet50 with only **23.5 M** parameters pretrained on the STL10 and ImageNet-1K datasets, as shown in Section 6.2. Then, the lower-complexity distilled model is used for downstream tasks such as classification.

## 6. Results

In this section, we demonstrate the performance of the proposed method for two downstream tasks: (a) SSL-based classification and (b) knowledge distillation-based classification.

### 6.1. SSL-Based Classification

We evaluate the representations obtained after pretraining the ResNet50 backbone with MV–MR on the ImageNet-1K and STL10 datasets for 1000 epochs using the loss function described above. The model pretrained on ImageNet-1K is evaluated with a linear protocol and a semi-supervised protocol with 1% and 10% of images labeled.

#### 6.1.1. Evaluation on ImageNet-1K

**Linear evaluation protocol**: A linear classifier is trained on top of the frozen representations of the ResNet50 [12] pretrained using MV–MR for 100 epochs with the cross-entropy loss.

**Semi-supervised evaluation protocol**: The pretrained ResNet50 is fine-tuned with a fraction of the ImageNet-1K dataset—1% or 10% of sampled labels for 100 epochs with the cross-entropy loss.

The results on the validation set of ImageNet-1K for linear and semi-supervised evaluation protocols of the model are shown in Table 1. The main advantage of the MV–MR is that it presents a new way to regularize latent space for self-supervised pretraining by using distance correlation between the embeddings from the model and hand-crafted image features. Due to the lack of computational resources, we did not run the parameter optimization for ImageNet-1K pretraining, so we think that the results could be further improved.

**Table 1:** **Classification accuracy. Evaluation on ImageNet-1K**. Evaluation of the representations from ResNet50 **non-contrastive** backbones pretrained with MV–MR on (1) linear evaluation protocol on top of frozen representations from ImageNet; (2) semi-supervised classification on top of fine-tuned representations, with 1% and 10% of ImageNet samples labeled. Top-1 refers to the accuracy of a classifier by determining if the highest-probability prediction is correct, and Top-5 refers to whether the correct answer is among the five highest probability predictions.

Method	Linear		Semi-Supervised			
	**Top 1**	**Top 5**	**Top 1**		**Top 5**	
			**1%**	**10%**	**1%**	**10%**
Supervised	76.5	-	25.4	56.4	48.4	80.4
PIRL [45]	63.6	-	-	-	-	-
SimSiam [6]	71.3	-	-	-	-	-
InfoMin Aug [46]	73.0	91.1	-	-	-	-
OBoW [47]	73.8	-	-	-	-	-
BYOL [37]	74.3	91.6	53.2	68.8	78.4	89.0
Barlow Twins [7]	73.2	91.0	55.0	69.7	79.2	89.3
VICReg [11]	73.2	91.1	54.8	69.5	79.4	89.5
**MV–MR (ours) **	**74.5**	**92.1**	**56.1**	**69.9**	**79.4**	**89.5**

##### Evaluation of Small Datasets

In this study, we demonstrate the self-supervised learning model performance on small-scale datasets. The model is trained on the STL10 and CIFAR20 [16] datasets with hand-crafted features: (i) flattened original images, (ii) augmented images, (iii) ScatNet features, (iv) HOG features, and (v) LSD features. The proposed model achieves state-of-the-art results in the linear evaluation protocol on the STL10 and CIFAR20 datasets compared to all other self-supervised methods. The results for STL10 are reported in Table 2, and those for CIFAR20 are in Table 3.

**Table 2:** **Evaluation on STL10**. Classification accuracy for the linear evaluation protocol on top of frozen representations from the STL10 dataset.

Method	STL10
ADC [48]	53.0
IIC [49]	61
TSUK [50]	66.5
SCAN [16]	80.9
ScatSimCLR [30]	85.1
RUC [51]	86.7
**MV–MR (ours) **	**89.7 **

##### Transfer Learning

To evaluate the pretrained representation of multiclass classification on the VOC07 [53] dataset, we train a linear classifier on top of the frozen representations from the pretrained encoder for 100 epochs. The mAP on the VOC07 dataset is reported in Table 4, along with results from other non-contrastive state-of-the-art SSL methods with a ResNet50 backbone.

### 6.2. Knowledge Distillation-Based Classification

To evaluate the proposed approach on the knowledge distillation-based classification task, we have used a pretrained CLIP [13] model based on the ViT-B-16 [14] encoder as the teacher and ResNet50 [12] as the student model. The CLIP model is trained based on the contrastive loss between the image and text embeddings. To proceed with the knowledge distillation in the same way as the SSL training, we use the default projector 8192-8192-8192 after the ResNet50 encoder. The pretrained CLIP ViT model uses images of shape 224×224×3 as an input and outputs a latent vector of shape 512, as shown in Figure 2. When reporting the results, the teacher model is evaluated using zero-shot evaluation on the ImageNet-1k dataset and a linear evaluation pipeline on other datasets. The student model is evaluated using a linear evaluation pipeline on all datasets.

The goal of the experimental validation is to demonstrate whether the ResNet50 model with 23.5 M parameters trained only on a smaller dataset can provide similar performance to the CLIP based on the ViT model with 86.2 M parameters and trained on 400 M images. It is important to point out that the training is performed without any additional labels, according to the proposed knowledge distillation framework. In Table 5, we report results of knowledge distillation, where CLIP based on ViT-B-16 is used as a teacher model and ResNet50 is used as a student model. The model is trained for only 200 epochs on a single NVIDIA RTX2080ti GPU using the proposed knowledge distillation approach on the STL10, CIFAR100, and ImageNet-1K datasets. The obtained results confirm that the convolutional ResNet50 model with 4× fewer parameters in comparison to the transformer ViT teacher model and trained on a considerably smaller amount of unlabeled data can closely approach the performance of the teacher model without any special labeling, clustering, additional augmentations, or complex contrastive losses. Remarkably, the proposed knowledge distillation largely preserved this performance and achieved 95.6% versus the best SSL MV–MR result of 89.7%, as indicated in Table 2 and Table 5. The proposed distillation method outperforms all other distillation methods on the CIFAR100 dataset: **78.6%** vs. current state-of-the-art 78.08% [54]. Thus, both the proposed MV–MR SSL training and knowledge distillation achieve state-of-the-art results on the STL10 and CIFAR100 datasets and demonstrate competitive results for the ImageNet-1K among all non-contrastive and clustering-free SSL methods.

### 6.3. Ablation Studies

In this subsection, we describe the ablation studies on the proposed losses (Table 6). In each of the experiments, we use the same training and evaluation setup: dataset—STL10, epochs—100, batch size—64, 16-bit precision, batch accumulation—1 batch. We use a linear evaluation pipeline. We demonstrate the impact of representation learning based on the maximization of the considered upper bound on the mutual information and the maximization of distance covariance in various settings. In this ablation, we show that the best results are achieved when using three loss terms: $L_{1}$, $L_{2}$, and $L_{3}$ (Appendix A).

## 7. Implementation Details

The architecture of the MV–MR is similar to ones used in other SSL methods such as BarlowTwins [7], VICReg [11], and others. The model $f_{\phi_{z}}$, shown in Figure 1, consists of two main parts: (i) the encoder, which is used for downstream tasks, and (ii) the projector, which is used for the mapping of encoder outputs to the embeddings used for the training loss functions in (Figure 1). In our experiments, we use standard ResNet50 [12], available in the torchvision library [55], as the encoder and projector, which consists of two linear layers of size 8192, followed by batch normalization, ReLU, and output linear layer.

We use computer vision feature-extraction methods applied to the original data: original RGB image (that is being flattened into a feature vector), ScatNet features of the image [56], randomly augmented images, flattening into a feature vector, histogram of oriented gradients (HOG), and local standard deviation filter (LSD filter) [20].

**ScatNet transform**: ScatNet [19,56] is a class of Convolutional Neural Networks (CNNs) that have a set of useful properties: (i) deformation stability, (ii) fixed weights, (iii) sparse representations, (iv) interpretable representation.

**Randomly augmented image**: In our experiments, we have applied the following augmentations to the image: random cropping, horizontal flipping, random color augmentations, grayscale, and Gaussian blur. Then, the image is flattened into a one-dimensional feature vector.

**HOG**: Histogram of oriented gradients (HOG) [21] is a feature description that is based on the counting of occurrences of gradient orientation in the localized portion of an image.

**LSD filter**: A local standard deviation filter [20] is a filter that computes a standard deviation in a defined image region over the image. The region is usually of a rectangular shape of size 3×3 or 5×5 pixels.

We use the PyTorch framework [55] for the implementation of the proposed approach. We use ScatNet with the following parameters: J=2 and L=8. We use the HOG feature extractor with the following parameters: number of bins—24 and pool size—8. We use a kernel of size 3×3 in the STD filter. As augmentations, for both image representation and as the input to the encoder, we use randomly resized cropping; random horizontal flipping with probability 0.5; random color-jittering augmentation with brightness 0.8, contrast 0.8, saturation 0.8, hue 0.2, and probability 0.8; random grayscale with probability 0.2; and Gaussian blur with a kernel size of 0.1 of the image size, mean 0, and sigma in the range [0.1,2].

For the losses, the margin parameter γ is set to 1, and ϵ is set to $1\times 10^{-4}$ in (7).

During the **self-supervised pretraining** experiments that are presented in Table 1 and Table 2, we train models for 1000 epochs, with batch size 256, gradient accumulation every 4 steps, base learning rate $1\times 10^{-4}$, Adam [57] optimizer, cosine learning rate schedule, and 16-bit precision. During **linear evaluation**, we train a single-layer linear model for 100 epochs with batch size 256, learning rate $1\times 10^{-4}$, and Adam optimizer. During **semi-supervised evaluation** on ImageNet-1K, we train a model for 100 epochs with batch size 128, learning rate $1\times 10^{-4}$, and Adam optimizer. During the knowledge distillation, we train the model for 200 epochs, with batch size 512, base learning rate $1\times 10^{-4}$, Adam optimizer, cosine learning rate schedule, and 16-bit precision.

When training, weight parameters λ=1 and μ=1 in in $L_{1}$, α=1 in $L_{2}$, and $\beta_{k}=1,k=1\ldots K$ in $L_{3}$.

## 8. Conclusions

In this paper, we introduce novel self-supervised MV–MR learning and knowledge distillation approaches, which are based on the maximization of several dependency measures between two embeddings obtained from views with and without augmentations and multiple representations extracted from non-augmented views. The proposed methods use an upper bound on mutual information and a distance correlation for the dependence estimation for the representations of different dimensions. We explain the intuition behind the proposed method of upper bound on the mutual information and the usage of distance correlation as a dependence measure. Our method achieves state-of-the-art self-supervised classification on the STL10 and CIFAR20 datasets and comparable state-of-the-art results on ImageNet-1K datasets in linear evaluation and semi-supervised evaluations. We show that ResNet50 pretrained using knowledge distillation on CLIP ViT-B-16 achieves comparable performance with far fewer parameters (23.5 M with ResNet50 vs. 86.2 M parameters with CLIP ViT-B-16) and a relatively small training set on multiple datasets: STL10 and ImageNet-1k. The proposed disillation method also achieves state-of-the-art peformance on the CIFAR100 dataset, 78.6% vs. previous state-of-the-art of 78.08%.

In our paper, we exclusively focus on the image-classification downstream task using ResNet architecture, limiting ourselves to its standard augmentation techniques. Future efforts should extend beyond ResNets to encompass transformers and other advanced deep learning architectures, exploring their applicability not just in classification but also in other vision downstream tasks, such as object-detection and -segmentation tasks, by using the pretrained backbone with a proper head that is finetuned for the selected downstream task. This expansion would allow for a broader range of augmentation strategies, such as Masked Image Modeling (MIM), and provide insights into the performance of different hand-crafted features across various architectures, enhancing the versatility of self-supervised learning approaches in computer vision. The code is available at: github.com/vkinakh/mv-mr.

## Figures and Tables

**Table 3:** **Evaluation on CIFAR20**. Classification accuracy for the linear evaluation protocol on top of frozen representations from the CIFAR20 dataset.

Method	CIFAR20
IIC [49]	25.7
TSUC [50]	35.3
SCAN [16]	50.7
RUC [51]	54.3
LFER Ensemble [52]	56.1
ScatSimCLR [30]	63.8
**MV–MR (ours) **	**73.2 **

**Table 4:** **Transfer learning on multiclass classification on the VOC07 [53] dataset**. Evaluation of the non-contrastive representations from the pretrained model on multiclass classification using the linear classifier on top of frozen representations. We report mAP.

Method	Linear Classification
	**VOC07**
Supervised	87.5
PIRL [45]	81.1
BYOL [37]	86.6
OBoW [47]	**89.3**
Barlow Twins [7]	86.2
VICReg [11]	86.6
**MV–MR(ours) **	87.1

**Table 5:** Knowledge distillation experiment with CLIP based on ViT-B-16 as teacher model and ResNet50 as a student model.

Approach	Parameters	STL10	ImageNet-1K	CIFAR100
CLIP ViT-B-16				
(zero-shot)	86.2 M	-	67.1	-
CLIP ViT-B-16				
(linear evaluation)	86.2 M	98.5	77.4	82.2
**MV–MR ** ResNet50				
(linear evaluation)	23.5 M	95.6	75.3	78.6

**Table 6:** **Ablation studies on the combination of losses**. We check the importance of each loss term for the training of the model. It is shown that using loss terms $L_{1}$, $L_{2}$, and $L_{3}$ provides the best classification performance. Also, we observe a phenomenon in which loss terms $L_{2}$ and $L_{3}$ work the best when applied jointly with the loss $L_{1}$. However, it is interesting to point out that a disjoint usage of these losses does not lead to reasonable performance enhancement. The exact nature of this phenomenon is not completely clear and additional investigation should be performed.

$L_{1}$	$L_{2}$	$L_{3}$	Accuracy	
			**Top 1**	**Top 5**
1 loss				
✓			50.86	93.95
	✓		46.71	92.18
		✓	44.1	92.08
2 losses				
✓	✓		50.76	93.83
✓		✓	47.39	92.54
	✓	✓	40.06	89.31
3 losses				
✓	✓	✓	**69.38 **	**98.85 **

## Data Availability

The datasets used in the papers are publicly available. The research code and pretrained models used for training and evaluation are publicly available.

## Abbreviations

The following abbreviations are used in this manuscript:

SSL	Self-supervised learning
MI	Mutual information
ViT	Vision transformer
CLIP	Contrastive language-image pretraining
MSE	Mean square error
LSD	Local standard deviation
HOG	Histogram of oriented gradients

## Appendix A. Ablation Studies Section 6.3

In this section, we describe the ablation studies on the combination of features for loss term $L_{3}$ (Table A1), a number of layers, and both the size of the projector in the trainable encoder (Table A3) and image augmentations (Table A2). In each of the experiments, we use the same training and evaluation setup: dataset—STL10 [15], epochs—100, batch size—64, 16-bit precision, batch accumulation—1 batch. When pretraining, all three loss terms are used. After model pretraining, it is evaluated using linear evaluation.

We describe the ablation studies on the combinations of features used for the $L_{3}$ loss term in combination with loss terms $L_{1}$ and $L_{2}$ in Table A1. We study the impact of features on the classification accuracy of the model. We use the following features in the study: the original image flattened into a vector, ScatNet [56] features of the original image, an augmented image flattened into a vector, a histogram of oriented gradients of the original image, and features from the local standard deviation filter (LSD). We use ScatNet with the following parameters: J=2 and L=8. We use the HOG [21] feature extractor with the following parameters: number of bins—24 and pool size—8. A kernel of size 3×3 is used in the LSD filter [20]. As augmentations for image representation, we use randomly resized cropping; random horizontal flipping with probability 0.5; random color-jittering augmentation with brightness 0.8, contrast 0.8, saturation 0.8, hue 0.2, and probability 0.8; random grayscale with probability 0.2; and Gaussian blur with a kernel size of 0.1 of the image size, mean 0, and sigma in the range [0.1,2]. We show that the best results are achieved when we use the combination of all feature extractors mentioned above.

The ablation studies on image augmentations are presented in Table A2. As augmentations, we compare randomly resized cropping, random horizontal flipping, random color augmentations, random grayscale, and random Gaussian blur. We use the same parameters for each augmentation as when augmented images are used as features. We show that the best classification results are achieved when a combination of random cropping, horizontal flipping, color jittering, and random grayscale is used.

The ablation studies on the number of layers and their size in the encoder’s projects are presented in Table A3. When the number of layers is larger than one in the projector, it consists of blocks with linear layers, batch normalization, and ReLU activation. We always keep the last layer linear. We show that the best classification results are observed when the projector consists of three layers, each with 8192-8192-8192 neurons.

**Table A1:** Ablation studies of the combinations of features used for the $L_{3}$ loss. In this setup, all three losses are used: $L_{1}$, $L_{2}$, and $L_{3}$. ScatNet transformation of the original image. Augmented image—randomly augmented original image. HOG—histogram of oriented gradients, computed from original view. LSD—original view filtered with the local standard deviation filter. Since all of these features are images, they are flattened before computing distance correlation.

Original Image	ScatNet	Augmented Image	HOG	LSD	Accuracy	
					**Top 1**	**Top 5**
1 feature						
✓					58.82	96.81
	✓				54.12	95.23
		✓			63.51	97.81
			✓		54.15	95.26
				✓	53.94	95.44
2 features						
✓	✓				64.44	97.97
✓		✓			66.18	98.39
✓			✓		63	97.78
✓				✓	63.14	97.8
	✓	✓			63.3	97.78
	✓		✓		62.95	97.6
	✓			✓	59.41	96.78
		✓	✓		63.66	97.69
		✓		✓	60.21	96.8
			✓	✓	62.46	97.71
3 features						
✓	✓	✓			65.82	98.18
✓	✓		✓		65.52	97.97
✓	✓			✓	60.96	97.08
✓		✓	✓		65.11	98.12
✓		✓		✓	65.19	98
✓			✓	✓	65.37	98.29
	✓	✓	✓		65.45	98.18
	✓	✓		✓	64.35	97.93
	✓		✓	✓	60.63	97.1
		✓	✓	✓	64.9	98.08
4 features						
✓	✓	✓	✓		68.25	98.45
✓	✓	✓		✓	68.2	98.53
✓	✓		✓	✓	64.56	97.44
✓		✓	✓	✓	67.21	98.48
	✓	✓	✓	✓	67.05	98.22
5 features						
✓	✓	✓	✓	✓	**69.38 **	**98.85 **

**Table A2:** Ablation studies on the image augmentations.

Random Crop	Horizontal Flip	Color	Grayscale	Blur	Accuracy	
					**Top 1**	**Top 5**
✓					43.02	90.31
✓	✓				49.99	93.98
✓	✓	✓			50.58	93.76
✓	✓	✓	✓		**70.82 **	**98.96 **
✓	✓	✓	✓	✓	69.38	98.85

**Table A3:** Ablation studies on the projector. Projectors consist of blocks with linear layers, batch normalization, and ReLU activation. We always keep the last layer linear.

Projector Size	Accuracy
**8192-8192-8192 **	**69.38**
4096-4096-4096	51.90
2048-2048-2048	51.35
1024-1024-1021	51.86
512-512-512	49.40
256-256-256	49.02
8192-8192	49.90
4096-4096	48.81
2048-2048	48.66
1024-1024	48.73
512-512	48.06
256-256	48.07
8192	48.65
4096	48.51
2048	48.20
1024	47.61
512	46.11
256	47.17
without projector	16.70
